# Convolutional neural networks (CNNs): concepts and applications in pharmacogenomics

**DOI:** 10.1007/s11030-021-10225-3

**Published:** 2021-05-24

**Authors:** Joel Markus Vaz, S. Balaji

**Affiliations:** Department of Biotechnology, Manipal Institute of Technology, Manipal Academy of Higher Education, Manipal, Karnataka 576104 India

**Keywords:** Convolutional neural networks, CNN, Pharmacogenomics, One-dimensional data, SMILES

## Abstract

Convolutional neural networks (CNNs) have been used to extract information from various datasets of different dimensions. This approach has led to accurate interpretations in several subfields of biological research, like pharmacogenomics, addressing issues previously faced by other computational methods. With the rising attention for personalized and precision medicine, scientists and clinicians have now turned to artificial intelligence systems to provide them with solutions for therapeutics development. CNNs have already provided valuable insights into biological data transformation. Due to the rise of interest in precision and personalized medicine, in this review, we have provided a brief overview of the possibilities of implementing CNNs as an effective tool for analyzing one-dimensional biological data, such as nucleotide and protein sequences, as well as small molecular data, e.g., simplified molecular-input line-entry specification, InChI, binary fingerprints, etc., to categorize the models based on their objective and also highlight various challenges. The review is organized into specific research domains that participate in pharmacogenomics for a more comprehensive understanding. Furthermore, the future intentions of deep learning are outlined.

## Introduction

The massive accumulation of data from genomics, transcriptomics, proteomics, metabolomics, and drug discovery has shifted the focus of ‘omics’ to ‘informatics’ due to the emergence of overwhelming biological data, referred to as the ‘Bigdata’, primarily arising from the high-throughput sequencing technologies [1, 2]. The rate of accumulation of new sequence data is far beyond the scientific communities’ capacity to determine their attributes through experimental methods [3]. Thus, a considerable amount of data is available to be processed and interpreted, and with significant improvements in computational resources, the time required to process a substantial amount of data has been dramatically reduced [4]. While conventional learning algorithms are inadequate in processing data present in its natural form, deep learning has brought about developments in solving problems in artificial intelligence [5]. These deep learning models have overtaken machine learning algorithms as they can extract features automatically; however, it also leaves the need for better model management for reproducibility as much information is lost during the model training [6]. Still, deep learning systems have defined structures and algorithms that allow them to learn through training, extract features, and alter the parameters to map the input and predict the output [7].

The idea of deep learning and neural networks has emerged from mimicking the neurons of the human nervous system. These artificial neural networks (ANNs) rapidly accept inputs and produce outputs with a complex network of neurons (hidden layers) for processing. The input nodes receive inputs and try to identify the nonlinear input–output relationship to calculate an optimal solution for the given problem [7]. The architecture of ANNs can be classified further as convolutional neural networks (CNNs) and recurrent neural networks (RNNs). The CNNs are a subset of ANNs, with each node detecting local features from the input vector, minimizing the parameters in a process called down-sampling, and the subsequent layers combining these features into a fully connected layer [8]. The RNNs, contrarily, are networks that show dynamic behavior by learning temporal tasks [9]. These models can input several data types, ranging from simple one-dimensional data to multi-dimensional data.

With these advancements, several new databases can be created to extract valuable information from the accumulated biological data. The applications of neural networks for processing such data present opportunities in genomics and drug discovery [2]. Deep learning strategies have made immense progress in recent years and gained popularity in handling huge volumes of structured data, widely used for processing images [10–12]. However, their implications in analyzing one-dimensional data like biological sequence data, SMILES codes, or medical texts are not widely recognized [13–16]. Biological sequences like DNA, RNA, or protein sequences can be identified as simple one-dimensional data that characterize a biological system, while SMILES data can depict chemical compounds. The potential benefits of deep learning and their success in data analysis are presented here. This could persuade biologists to join the community, learn, and develop more models on their own in their respective fields of study.

**Fig.1:**
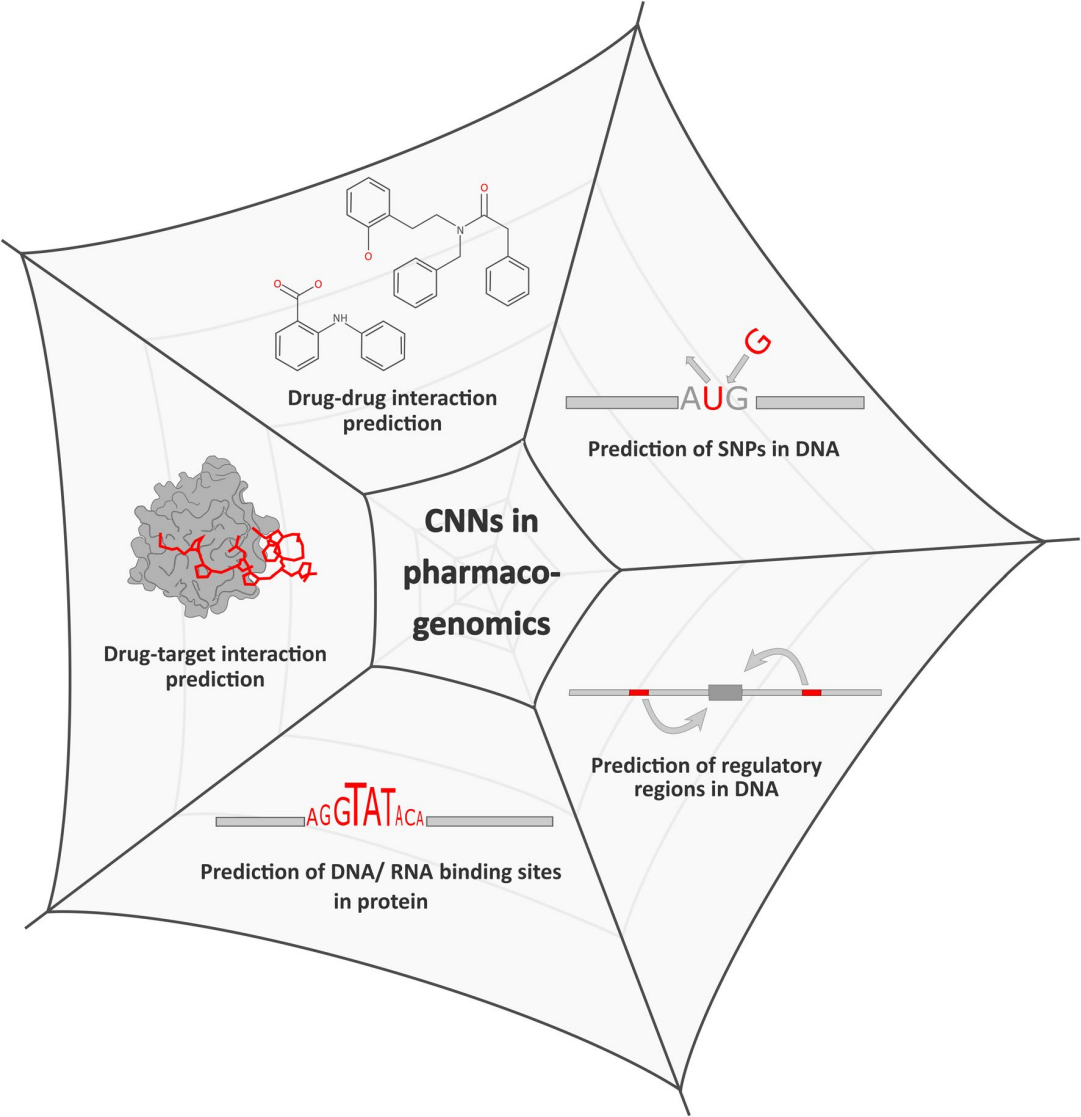
Classification of CNN methods into five major subdomains, each corresponding to the final objective of the analysis

In this review, we elucidate CNNs as a valuable tool in pharmacogenomics for biological data analytics. As most biologists are not specialized in machine learning, we aim to introduce deep learning methods, specifically CNNs, to analyze one-dimensional data. In this regard, various CNN methods integrated with other machine learning algorithms developed in the past decade are reviewed. An outline of essential components of a CNN is presented along with its applications. The applications of these strategies are broadly categorized into five subsections as follows: (1) prediction of single nucleotide polymorphisms (SNPs) in DNA, (2) prediction of regulatory regions in DNA, (3) prediction of DNA/RNA binding sites in proteins, (4) prediction of drug–target interactions, and (5) prediction of drug–drug interactions, as illustrated in Fig. 1.

## Overview of CNNs

**Fig.2:**
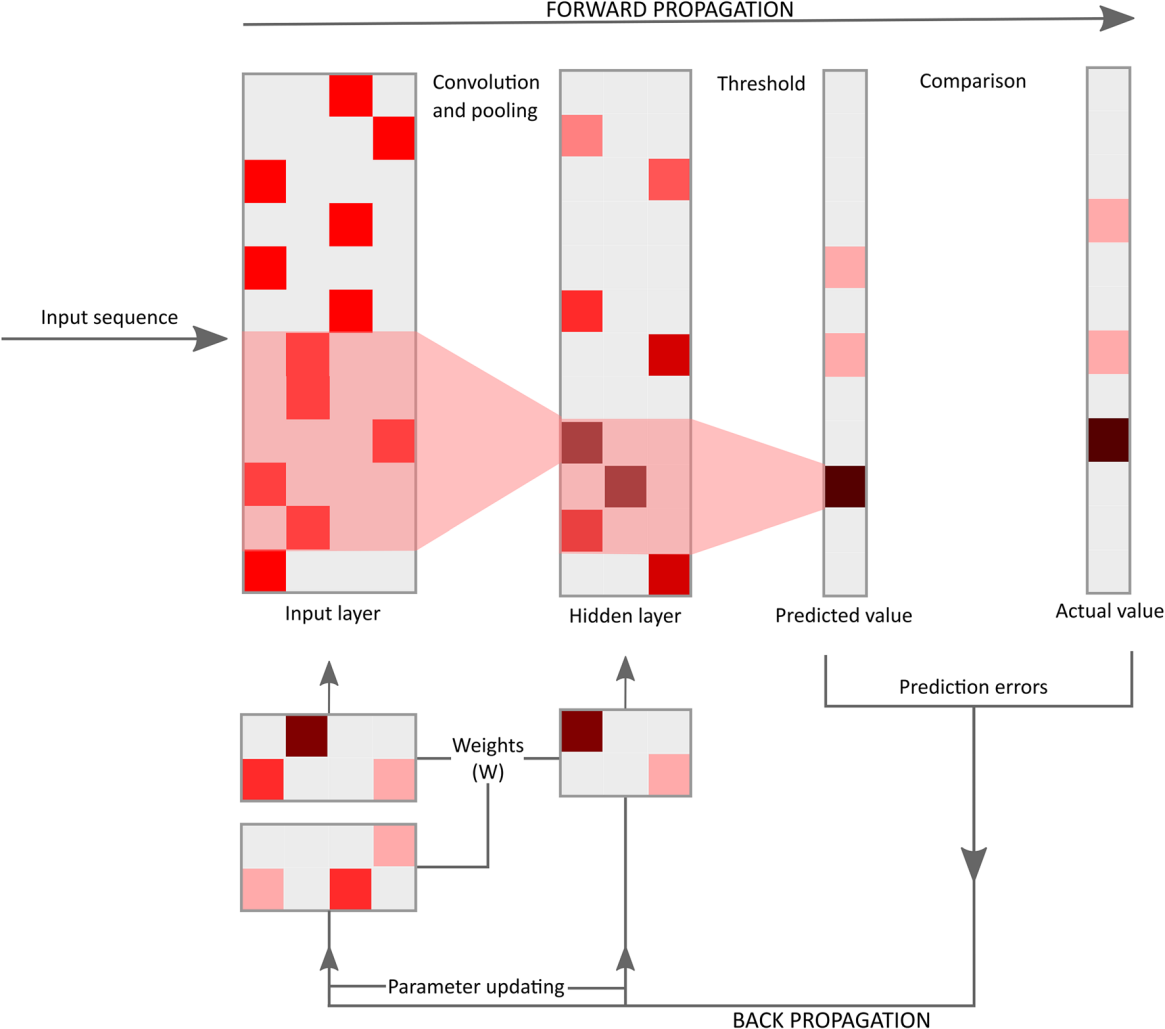
Basic architecture of a CNN. The input layer extracts information from the input sequence by multiplying with weights. The subsequent layers perform the function of convolution and pooling, wherein these layers extract local information and pool it, reducing dimensions of the sequence vector. Fully connected layers have its nodes connected to all the nodes in the previous layer. The final activation function outputs the sequence classification. This predicted value is compared to the actual annotated value when the model is being trained. The prediction errors are assessed, and the model undergoes back propagation iteratively to update the existing parameters each time to reduce the errors in prediction until the values converge

CNNs are a collection of neurons that are organized in interconnected layers, with convolutional, pooling, and fully connected layers [17]. As a mathematical construct that processes data of multiple dimensions, CNNs are designed to adaptively learn simpler patterns at lower depths while transitioning to more complicated patterns as we dive deeper. Deep neural networks overcome the use of exponentially large parameters by the addition of multiple hidden layers. There are two significant characteristics of a CNN: weight sharing and local connectivity [18]. Weight sharing implies uniform weights across the nodes in the layer. Local connectivity is the term used when each node receives input only from a few local values in an array, and each output is related to only certain parts of the input vector (Fig. 2).

### The CNN framework

The convolutional layers perform operations of convolution and activation. A linear operation like convolution is where each of its layers performs an element-wise multiplication between an array of features called a kernel and the input of array numbers called a tensor [19]. The kernel is usually of a defined size, 3 × 3 or 5 × 5. The repeated operations on smaller arrays in local patches that make up a single array give rise to a feature map, which acts as an input to the next layer in the network. Such persistent operations at several locations detect local conjunctions from the layer [20]. A convolutional layer with ‘n’ kernels can detect ‘n’ local features that result in the formation of ‘n’ feature maps [21]. A subsequent activation function follows, which computes the function’s nonlinearity, a jump from previously conducted linear convolution operations, with the commonly applied nonlinear functions being the tangent function (tanh) or the rectified linear unit (ReLU).

The pooling layer reduces the dimensions of the input layers, subsequently reducing layer parameters. This operation merges similar features by shifting the patches containing these features across rows or columns [5]. While a more commonly used pooling method, max-pooling, selects the highest value out of a kernel to pass through to the output tensor, average pooling calculates the average in the kernel [22, 23].

Fully connected layers connect every local input from the previous layer to every output in the next layer. Its role is to combine portions of the generated outputs from convolutional layers with a one-dimensional vector consisting of probabilities of each feature belonging to a label. Nodes in these layers have a learnable weight that can map inputs to the desired outputs [24]. The final layer consists of an activation function different from the other layers, with softmax as an example for classification problems.

### Training a neural network

Training a neural network is the process of finding optimal weights and biases for nodes in a layer. In CNNs, training aims at finding optimal kernels in convolutional layers and weights in the fully connected layers [25]. Forward propagation inspects input features from the previous layer and produces an output across the hidden units first and then to the output layer with a final nonlinear activation function. The task involves initializing parameters, kernels, and weights with random values with the input of feature vectors from the training dataset to obtain the corresponding output value for every node in each layer. To calculate errors at each output, the loss function is evaluated to check for model performance. Backpropagation involves the optimization of algorithms by changing parameters in each node using gradient descent. For each weight, the gradient descent is the deviation in the amount of loss when that weight is altered by a small amount. After repeated iterations, it eventually calculates the optimal parameters that provide minimum loss in the algorithm [26].

**Fig.3:**
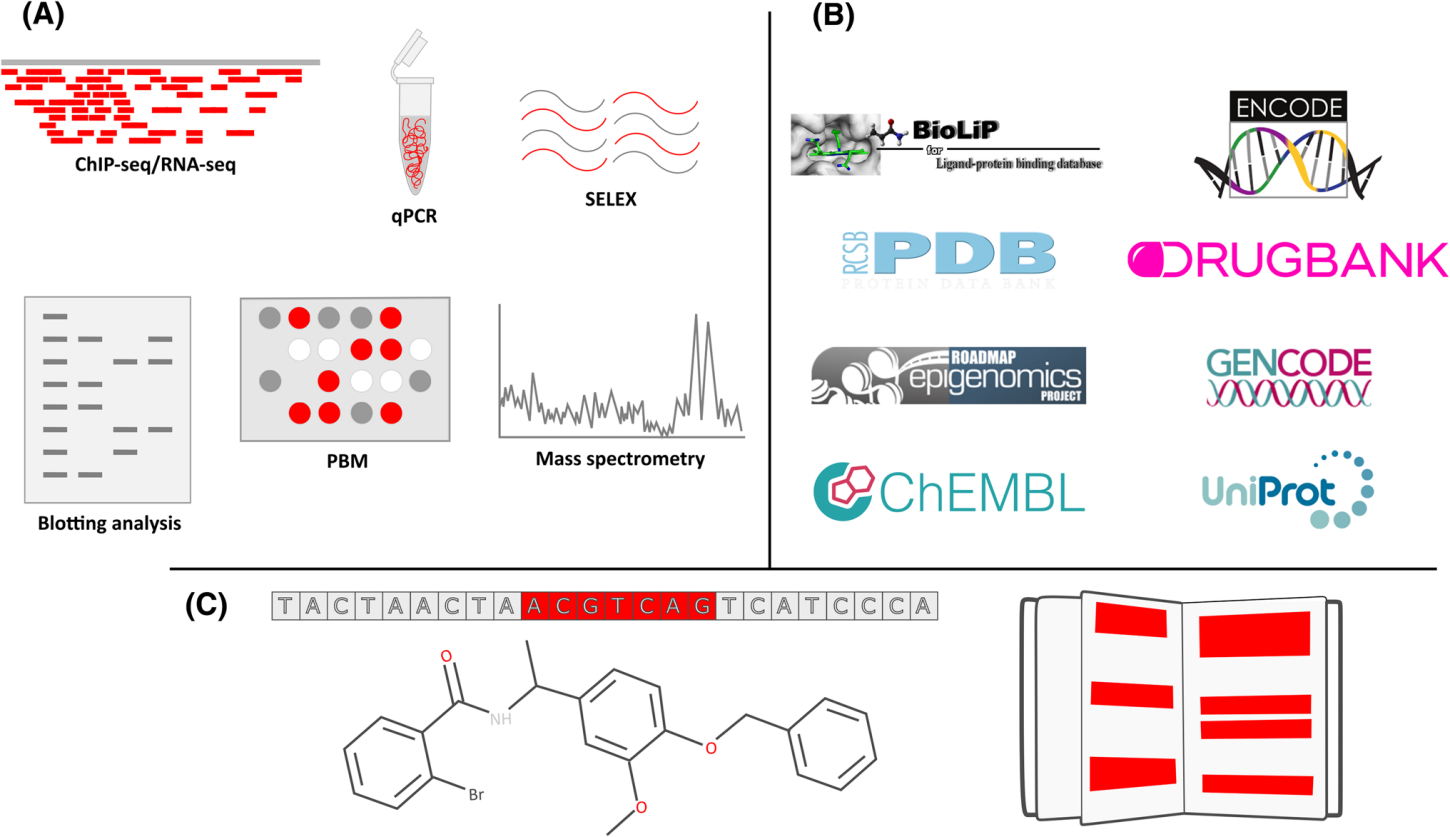
The production of datasets. (**a**) Different techniques involved to create annotations on the sequences. Some of these techniques include ChIP-seq to identify protein binding sites, mass spectrometry to identify protein/drug structures or qPCR to quantify the gene expression. (**b**) Annotated sequences, SMILES codes or interaction networks uploaded to various databases like Protein Data Bank (PDB), DrugBank, or large-scale projects like ENCODE, Roadmap Epigenomics. (**c**) Obtained annotated sequences, SMILES representation present in databases or medical texts containing unstructured data of drug–target or drug–drug interactions

Data collection can be from various sources such as public repositories, clinical reports, experimental or synthetic datasets, depending on the requirements (Fig. 3). For instance, DeepECA, a model predicting protein contact from multiple sequence alignment, obtained the 1D amino acid sequence data using PISCES, a PDB sequence culling server [27]. Similarly, for DNA-binding protein identification, Shadab et al. extracted information from Protein Data Bank (PDB) and named the training dataset as ‘PDB1075′ [28]. Training a deep CNN from scratch has its challenges. A deep CNN requires large amounts of medical data, but diseases that occur less frequently tend to have smaller datasets. Poor memory and computational resources with complications due to overfitting require a great deal of patience and expertise [29]. An alternative to this would be to implement pre-trained CNNs and fine-tuned according to the application [30, 31]. To increase the performance with smaller datasets, any of the following strategies can be used, such as data augmentation [32], transfer learning [33], and capsule network [34].

### Hyper-parameter and parameter tuning

A parameter is a variable that is interior to the model and configured by learning the data. Parameters can only be initialized but are not set by the user, and it determines the performance of the model, for instance, kernel and weights. Hyperparameters are set by the user and are external to the model. These include learning rates, number of iterations, and number of layers. Tuning involves collecting weights of the layers from previously trained models to a new network, except for the last fully connected layer [35]. Assessment of the required number of parameters and hyperparameters may vary according to the application [36]. The number of parameters required directly correlates to the complexity of the neural network, and it will have a significant impact on the accuracy [37]. Too many parameters can cause overfitting.

## Pharmacogenomic data analysis using CNNs

There is an increase in the availability of sources from where data can be extracted (Fig. 3). This data can be one-dimensional biological sequences, such as DNA, RNA, or protein sequences. For small molecules, data formats, such as SMILES, SMARTS, InChI, binary fingerprints, can be used to represent chemical structures. Besides, medical literature that includes text briefings about biomolecular targets and biomarkers is also one-dimensional. This data may not provide knowledge on prediction-based analysis unless processed in machine learning models. Likewise, machine learning models are ineffective without incorporating appropriate datasets. The following text reviews the relationship between prediction tools and learning data. We constrict the CNN approach as a prediction tool and one-dimensional input as learning data to summarize applications and improvements in pharmacogenomics prediction through recent years. For biological sequences, we have classified pharmacogenomics analysis into the prediction of SNPs in DNA, prediction of regulatory regions in DNA, and prediction of DNA/RNA binding sites in proteins; we have involved SMILES representation in drug–target interaction prediction and lastly, medical texts in drug–drug interaction prediction. A summary of all the models communicated in this article is provided in Table 1.

**Table 1:** A summary of CNN models along with the applications and challenges

Sl No	Model name	Description	Applications	Dataset (*DS*)/Database name (DN)/Source method (SM)	Challenges addressed	References
1	DeepSEA	3 layers with kernel numbers 320, 480 and 960, respectively	Predicting effects of non-coding variants, transcription factor binding, DNase I sensitivity, and histone marks	*DB:* ENCODE and Roadmap Epigenomics data	Achieving single-nucleotide sensitivity; Flexibility in the model to address more complex mechanisms involved	[38]
2	DeepVariant	Learning rate 0.0015; momentum of 0.8 and the output layer being a three-class Softmax classifier	Variant calling in sequencing technologies	*DS:* NA12878 *DB:* Platinum Genomes Project	Manual adjustment of features in statistical models, assumption that the read errors are independent	[15]
3	NeuSomatic	9 convolutional layers; initial learning rate and momentum of 0.01 and 0.9, respectively	Identification of the length and type of a somatic mutation	*DS:* Mixture of NA12877 and NA12878, ICGC-TCGA DREAM challenge dataset, PacBio dataset and real datasets, CLL1 and COLO-829	Achieved the best accuracy when compared to all the other tested models across multiple datasets for different tumor purities	[39]
4	Cancer classification	2 hidden layers with sigmoid activation function in the output layer	Classification of Leukemia, Adenocarcinoma, Breast cancer, ovarian cancer	*DS:* Cancer datasets from various papers	Data insufficiency problem	[40]
5	Detecting SNP sites	Bi-stream CNN model with 8 hidden layers that includes 4 convolutional layers; learning rate 0.01 and momentum 0.9; final fully connected layer consists of 512 nodes	Applied to datasets with human Down Syndrome samples	*DB:* Illumina exome data	Limited number of machine learning algorithms available for human Down Syndrome studies	[41]
6	Deopen	CNN-three-layer FNN hybrid; filter size 20 and learning rate 0.7	Prediction of chromatin accessibility and identification of functionally influencing SNPs	*SM:* DNase-seq experiments *DB:* ENCODE project	Greater ability to capture regulatory codes of DNA, potential to identify the impact of non-coding variants on gene expression	[42]
7	Identification of the conserved sequence motifs	-	Applied to enhancers across different mammalian species	*DB:* UCSC Genome Browser, Ensembl v73	Generalizing the model for all species while being trained for only a single species	[43]
8	iEnhancer-ECNN	6 convolutional layers and two fully connected layer with 768 and 256 nodes; 0.0001 learning and 20 epochs	Prediction of enhancers	*DS:* Enhancer sample dataset obtained from other authors	Low Matthews correlation coefficients (MCCs)	[44]
9	BiREN	3 convolutional layers with the first consisting of 320 kernels; 925 nodes in fully connected layer	Prediction of enhancers	*DB:* VISTA Enhancer Browser, UCSC Genome Browser, ENCODE, Roadmap Epigenomics Project	Limited availability of enhancer data	[45]
10	DeepEnhancer	4 convolutional layers with the first containing 128 kernels of size 1 × 8; Final fully connected layer of 128 nodes; 0.5 dropout rate; learning rate as 0.0001 and 30 epochs	Prediction of enhancers	*DB:* Enhancer databases from FANTOM5 and ENCODE	Failure to record sophisticated features from enhancer sequences	[46]
11	CNNProm	1 convolutional layer with 200 filters; fully connected layer of 128 nodes; 5 epochs	Classification of promoter sequences, given RNA samples	*DB:* EPD	Poorly recorded universal characteristics of promoters	[47]
12	DeeReCT-PromID	2 convolutional layers with filter length 15; dropout rate 0.5	Identifying RNA polymerase II core promoters in human RNA sequences	*DB:* EPDnew	Learning patterns for longer input sequences	[48]
13	Xpresso	2 convolutional and fully connected layers; 10 epochs and a dropout rate of 0.5	Evaluation of mRNA expression levels	*SM:* RNA-seq *DB:* Roadmap Epigenomics project	The degree to which promoter sequences influence gene expression levels was unanswered	[36]
14	DNA binding site prediction	4 convolutional layers with filter sizes 9 × 1 and 7 × 1; Run for 100 iterations	DNA–protein binding sites datasets	*DS:* PDNA-543, PDNA-224, PDNA-316	Improved sensitivity, specificity, and accuracy than the models compared alongside	[49]
15	DeepBind	Motif lengths of 14, 20, 24, 32; learning rate and momentum in the ranges 0.0005–0.5 and 0.95–0.99, respectively	Identification of DNA-/RNA- binding sites; examination of SNVs in promoters	*SM:* In vivo ChIP-seq, CLIP-seq, RIP-seq; HT-SELEX data	Applied to microarray and sequencing data; toleration of noise and mislabeled data; Automatic calibration of the parameter	[50]
16	DeepDBP-CNN	Convolutional layer uses 128 filters of size L × 31 to extract 128 feature maps (L is the length of the vector)	Identifying DNA-binding proteins	*DS:* PDB186	Manual feature extraction from other models	[28]
17	iDeepE	2 layers of convolutional, max pooling and fully connected layers; filter length 16 and learning rates 0.001 and 0.0001	RNA binding protein (RPB) binding site prediction	*DS:* RBP-24, RBP-47 *SM:* CLIP-seq	Extracting crucial information from local sequences	[51]
18	iDeepS	Epoch set as 30 and filter length 10	RBP binding site prediction	*SM:* CLIP-seq	Detection of sites in structure motifs was not possible in iDeepE	[52]
19	Calculation of K_D_ values	3 hidden layers with 12-nucleotide k-mer	Identification of miRNA target sites	*DS:* miRNA-transfection datasets	Calculation of the relative K_D_ for sequences of length ≤ 12 nucleotides	[53]
20	QSAR model	2 convolutional layers, 5 max pooling layers, 2 fully connected layers	Identifying chemical molecules that target a given protein	*DB:* PubChem database	SMILES codes can be represented as fixed-size features	[54]
21	FP2VEC CNN	1 convolutional layer, max pooling, fully connected layer each; dropout rate 0.5	QSAR model to predict the biological activity and properties of chemical compounds	*DS:* Tox21, HIV, BBBP SIDER, Malaria, CEP, ESOL, FreeSolv and Lipophilicity	Fast and training, high accuracy and effective as a multitask learning method	[55]
22	DeepACTION	Learning rate of 0.0001; 1483-dimensional feature vector	DTI prediction model	*DB:* DrugBank, KEGG	Integrated MMIB to handle imbalanced datasets and LASSO for high-dimensional data	[56]
23	Transformer-CNN	100 epochs; learning rate 0.001; < 100 iterations	QSAR model to predict the biological activity and properties of chemical compounds	*DB:* ChEMBL database	No adjustable parameters, so less overfitting	[57]
24	DeepDTA	2 CNN blocks, each with 3 convolutional layers, 1 max pooling layer, 3 fully connected layers; dropout rate 0.1; learning rate of 0.001; 100 epochs	PCM model to predict drug–target interactions	*DS:* Davies Kinase dataset and KIBA dataset	Produces better accuracy with only raw sequences of compounds than methods that included structural data	[58]
25	FRnet-DTI	FRnet-Encode: 2 fully connected layers Learning rate of 0.001; Dropout rate of 0.5	Two model architecture for DTI; FRnet-Encode for feature extraction and FRnet-Predict for classification problem	*DB:* DrugBank, BRENDA, KEGG, SuperTarget	Boosted an improved accuracy, although not the best from the models tested	[59]
26	Attention-based multi- scale convolutional encoder	4 convolutional layers	Predicting drug sensitivity (IC_50_) values for a chemical compound	*DB:* GDSC database	Higher significance of results produced due to strict training and evaluation; the cells and compounds were split and did not see each other during training	[60]
27	DeepPurpose	-	DTI prediction model that uses CNN on SMILES strings	*DS:* DAVIS, KIBA	Availability of a web interface	[61]
28	ConvS2S	Learning rate 0.00001	Predicting compound's aqueous solubility	*DS:* Delaney aqueous solubility dataset	No structural data, or ‘engineered features’ that if present, limit the applicability of the model	[62]
29	DeepConv-DTI	Learning rate 0.0001; 15 epochs; dropout rate of 0	Detecting protein binding sites for drug–target interactions	*DB:* DrugBank, KEGG, and IUPHAR	Since protein structures are limited, an input of raw protein sequences provides a larger training dataset	[63]
30	DTI-CNN	1 of each convolutional, max-pooling and fully connected layers; Convolutional layer consisting of 4 kernels; Learning rate of 0.001, dropout rate 0.5 and 35 epochs	Constructing heterogeneous networks of protein and drugs for DTI prediction	*DB:* DrugBank, HPRD, Comparative Toxicogenomics Database	Dimensional reduction and improved accuracy	[64]
31	DDI extraction model	A ‘look-up’ table layer for position and word embedding representation; 3 hidden layers; dropout rate of 0.5	DDI extraction from medical literature	*DS:* DDI corpus of the 2013 DDIExtraction challenge, consisting of DrugBANK and MEDLINE data	First ever CNN model for DDI extraction, improved accuracy than other machine learning methods	[65]
32	Multi-channel CNN for DDI extraction	-	DDI extraction model consisting of multi-channels	*DB:* DrugBank, MEDLINE	Maximum coverage of sentences due to multi-channels	[66]
33	DDI extraction model	1 of each convolutional, max-pooling and fully connected layers; 200 filters of each window size; dropout rate of 0.5; maximum sentence length of 128; 27 epochs	DDI extraction without using any external features	*DB:* DrugBank, MEDLINE	No external features, hence, the improved reliability on the learning process	[67]
34	Two stage learning Bi-LSTM CNN model	1 of each convolutional, max-pooling and fully connected layers; 200 filters of each window size; dropout rate of 0.5, learning rate of 0.001	DDI extraction from English and Spanish medical texts	*DS:* DDI corpus of DDIExtraction, eHealth-KD challenge dataset	Outperformed complex CNN models of 10 layers; can be used on different languages	[68]
35	SGRU-CNN	1 of each convolutional, max-pooling and fully connected layers; maximum sentence length of 186; learning rate of 0.0005 and dropout rate of 0.8; feature vector dimensions: position embeddings as 50 and word embeddings as 300	DDI extraction from medical literature	*DS:* DDI corpus of the 2013 DDIExtraction challenge	No external features or any linguistic tools	[69]
36	AGCN	1 of each convolutional, max-pooling and fully connected layers; dropout rate of 0.5	DDI extraction from medical literature	*DS:* DDI corpus of the 2013 DDIExtraction challenge	A self-attention technique to ignore irrelevant information	[70]
37	RHCNN	An embedding layer used, similar to the ‘look-up’ table layer; 2 of each convolutional and max pooling layers; dropout rate of 0.5	DDI extraction from medical literature	*DS:* DDI corpus of the 2013 DDIExtraction challenge	Novel method of using dilated convolutions for the given dataset	[71]

### Prediction of single nucleotide polymorphisms (SNPs) in DNA

Mutations in the genomic sequences may lead to diseases and disorders. Interpreting these signatures is imperative for early detection and treatment. Although conducting biological experiments help record the gene expression data that infer the phenotypes or functions of cells, profiling such data for diseases is intricate due to the amount and complexity of the genes. On the other hand, characterizing SNPs has been challenging due to sensitivity issues, as modeling the functions requires a precise prediction of single-nucleotide sensitivity [38, 40]. Other setbacks faced by machine learning methods include the insufficiency of data from rare disorders, risk of overfitting, and difficulty integrating data samples from different gene expression platforms [40].

While CNNs are yet to be modeled as an ideal method, they have shown promises over other machine learning methods in certain frontiers. A framework to detect non-coding variants, DeepSEA, was developed by Zhou and Troyanskaya [38]. It was trained using transcription factor binding data; the position of the non-coding variant dictated its regulatory properties. This model can predict the influence of several SNPs on transcription factor binding. For instance, a ‘C to T’ mutation at SNP locus rs4784227 on the transcription factor FOXA1 induces the risk of breast cancer, and an SNP, ‘T to C’ at the binding site for GATA1 may lead to α-thalassemia. DeepVariant could detect indel variants in whole-genome sequencing (WGS) data and exome data with high sensitivity even after limiting the training dataset [15]. Unlike DeepVariant that uses read pileup as input, NeuSomatic functions with base frequency as the input data and detects somatic mutations using sequence alignment while dealing with greater accuracy [39]. NeuSomatic can predict the type and length of the somatic mutation and has a CNN structure inspired by ResNet [72]. Training this model on two real WGS datasets consisting of chronic lymphocytic leukemia and melanoma data obtained a test accuracy of > 99% and > 93%, respectively. This method was suggested for broader applications in somatic mutation detection. The model Basset predicted Genome-wide association studies (GWAS) SNPs that likely affected the local gene expression [73]. SNPs from GWAS were tested to interpret the relationship between genetics and bipolar disorder [74]. This model yielded a test accuracy of 91% and 92% and detected 137 and 407 risk genes, respectively, of which 22 and 51 genes were reported to be associated with the occurrence of bipolar disorder.

Multi-task deep learning (MTDL) algorithm was developed to classify different cancers [40]. The insufficiency of datasets in learning algorithms was solved using different gene features for the same output label on two evaluation sets (e.g., tasks involving acute myeloid leukemia as the output). In total, 12 tasks for evaluating its performance were used, such as adenocarcinoma, seminoma, ovarian cancer, and colon cancer. Feng et al. developed a bi-stream model that simultaneously inputs two SNP maps [41]. These maps were obtained by converting the SNP intensities at each site into chromosomal SNP maps at the initial stages. This model was established for predicting human Down Syndrome, a disorder of intellectual instability caused by genomic duplications and dosage imbalances, like microduplications at human chromosome 21.

### Prediction of regulatory regions in DNA

Variations in the gene expression levels can directly contribute to complex diseases; hence it is vital to understand DNA sequence components that constitute gene regulation. Prediction of the precise impact of such regulatory elements can help progress in diagnosis and medicine. A model like Deopen can read DNA regulatory codes and predicted chromatin accessibility [42]. Enhancers are sequences far from promoters that bind to the transcription factors to regulate gene expression, and these are critical for healthy cellular development and differentiation [75]. Exploring enhancers in sequences has led to a multi-layered CNN model proposed by Chen et al. to capture complex sequences [43]. Testing this model for different species inferred the conservation of these sequences across mammals. Other CNN models that predict enhancer sites are iEnhancer-ECNN [44], BiREN [45], and DeepEnhancer [46]. An ensemble learning algorithm consisting of CNNs was introduced in iEnhancer-ECNN. Analysis for the area under the receiver operating characteristics curve (AUC) and accuracy recorded higher values in iEnhancer-ECNN than in models such as iEnhancer-2L, EnhancerPred, and iEnhancer-EL. Learning enhancer elements using BiRen achieved high performance, with an AUC of 0.945. DeepEnhancer used datasets from the ENCODE and FANTOM5 project [76]. FANTOM5 consisted of maps of promoters and enhancers present in mammalian cell lines. Compared to the gapped k-mer support vector machine (gkmSVM), DeepEnhancer had a higher AUC [46].

Promoters are the regions in DNA that denote the start of transcription. The design principle of these sites is difficult as promoters are gene-specific, and hence the diversity is broad [77]. Designing computational methods here is challenging as sequence features from other models are hard to reuse. A few promoter site recognition models include CNNProm [47], PromID [78] and DeeReCT-PromID [48]. CNNProm was learned using a well-known promoter class, TATA promoters for eukaryotes present in the EPDnew promoter database, and sigma70 sub-class promoters of *E.coli*. PromID was an improved model that outperformed its predecessor, CNNProm, with improved precision and lesser likelihood to produce false positives. DeeReCT-PromID had a similar impact and could study longer sequences with higher precision.

The principle of ‘achieving mRNA abundance from recognizing promoter sequences in the genome’ was applied to predict the gene expression levels from the given sequence [36]. Several other attempts to record gene expression by correlating it to transcription factor binding have brought about issues in expected motif binding and signal identification, delivering unlikely false positives and noise in sequencing data [79]. Constructing new models that do not use such experimental data could bring about promises in regulatory mechanisms. In this experiment, the model accurately predicted the expression levels in genes of cells like human lymphoblastoid cells and human myelogenous leukemia cells. It was estimated from this model that promoter sequences cause ~ 50% of the gene expression variability. However, other aspects of gene expression remain undiscovered, potentially giving rise to more complex models in the future.

### Prediction of DNA/RNA binding sites in proteins

DNA binding proteins are the proteins that have a common DNA binding domain but a discrete sequence of amino acids that allow for specific binding interactions. Examples of DNA binding proteins include DNA polymerases, coactivators, corepressors. These are involved in several aspects of genetic activity like packing, replication, transcription, repair [80]. Genetic signals associated with them play a crucial role in gene expression and cell development that directly associates with studies in complex traits, the pathogenesis of diseases, and the characteristics of diseases like diabetes and cancer [81].

CNN models to identify specific protein sequences that bind to DNA have been developed alongside datasets like PDNA-543, PDNA-224, and PDNA-316 and have been used to evaluate performances of the features’ position-specific scoring matrix (PSSM), one-hot encoding, and predicted solvent accessibility (PSA), that further lead to the prediction of DNA binding sites in protein [49]. This model was a combination of features in a CNN, with an ensemble classifier. It obtained a test accuracy of ~ 90% on the dataset PDNA-543, higher than in predictor models TargetDNA and EC-RUS (WSRC). DeepBind was an upgrade from traditional scoring matrices and could be applied to microarray and sequencing data [50]. It was evaluated alongside 26 other algorithms [82] using protein binding microarray (PBM) data, and it outperformed all the other methods. A trick into presenting better learning algorithms is to follow the two rules given; reverse complementing the DNA strand and treating it as another sample; extending the DNA sequence, and dividing it into three shorter sequences [81]. This enabled the CNN model to understand the relationships between the double-strand DNA sequences better. This strategy applied to DeepSea [38], and DeepBind models significantly improved AUC. DeepDBP-CNN, inspired by previously existing models like DeepBind, used pre-learned embedding and CNN and produced a training accuracy of > 94%, a sensitivity of 0.83, and an AUC of 0.986 [28]. A comparison of DeepDBP-CNN with other methods showed promising results. An SVM classifier model like HMMBinder, trained with the same dataset (PDB 1075), had an accuracy of ~ 86%, a sensitivity of 0.87, and an AUC of 0.902, while other SVM-based models performed even more poorly. A useful tactic to prevent overfitting is to introduce a dropout layer at the end [83]; this layer will randomly drop a node with all its connections and hence make the model prevent overfitting to some degree.

RNA binding proteins (RBP) can recognize specific RNA sequences or structural patterns, called motifs. Like DBP, such proteins play a role in stability, cellular localization, and transport while associating themselves in co-transcriptional and post-transcriptional processes [84]. These motifs observed in RBPs can be obtained using in vitro assays like RNAcomplete [85]. With such findings differing in different cell environments and proving costly, an alternative approach was to apply deep learning, specifically CNN models using RNA primary sequence as an input to locate sequence binding motifs. A global module of iDeepE, iDeepE-G used techniques similar to that in DeepBind and RNA padding (extending all sequences to that of the longest available sequence) [52]. This module evaluated with the RBP-24 dataset had an average AUC of 0.931, and this model performed the best out of other sequence predictors like ResNet-E, Pse-SVM, GraphPlot, and Deepnet-rbp. A drawback of iDeepE is that it requires a broader training set to generate a better model. iDeepS, proposed by the same author [52], introduced the identification of structure binding motifs. Examples of binding discovery using the structure motifs by iDeepS included the preference of protein hnRNPC binding to U-rich hairpin structures and the interaction of protein PUM2 with UA-rich stem regions. A CNN model to predict enhancer-promoter interactions was developed by Zhuang et al., (2019), which performed as effectively as a complex CNN-RNN model hybrid [86]. Argonaute is a protein associated with the post-transcriptional regulator microRNA (miRNA) to form RNA-induced silencing complexes (RISC) [53]. This complex results in the silencing of gene expression and further mRNA degradation. McGeary et al. approached this prediction of repression with a model that calculated the K_d_ values for miRNA binding sites [87].

### Prediction of drug–target interactions

Drug–target interaction (DTI) prediction is essential for assessing interactions that lead to the identification of new drug candidates and can predict many of its side effects before the start of clinical trials [88]. In vivo techniques are expensive, and while they are accurate, the proposal of exploring every possible drug for a target seems laborious and tedious in practice [89]. Moreover, very few compounds worked on end up in the market as drugs after years of research, mainly due to their toxicity and side effects. In silico methods can narrow down these chemicals much quicker, making it feasible to experimentally work only on the shortlisted candidates. The fundamental idea in drug discovery is that chemically similar drugs interact with similar protein targets in our system. These predictions can be made from 3D protein structures using methods like ligand-based approaches that scan through databases to identify existing ligands that fit into a given receptor [90] or structure-based approaches that build ligands from small fragments of molecules binding to different locations in a target site [91]. In either of these methods, there is a requirement for obtaining the 3D structure of the protein and the ligand, and this a complex task as it is done through strenuous experimental processes. Hence, there is a requirement to shift toward methods that are simple and straightforward and use 1D data such as DNA/protein sequences and SMILES representations of small molecules. These datasets can be obtained from databases such as DrugBank, ChEMBL, STITCH, KEGG, for computational analysis to identify relationships between drug and target protein interactions and consequently predict new drugs that alter the disease state by regulating the activity of the molecular targets [56]. Validation of such targets follows that use in vitro or in vivo models.

The models that identify the relationship between the physicochemical properties of chemical structures and their biological activities are termed as Quantitative Structure–Activity Relationship (QSAR) models, and these aim at modeling the ligand descriptors [92, 93]. This is where we shift our focus away from genomic sequences and attempt to model the chemical compounds. Hu et al. used SMILES strings as an input to a CNN model for accurate QSAR prediction [54] and applied it to FP2VEC [55]. This deep learning method can identify the activities of small molecules. The molecular featurizer FP2VEC correlates chemical compounds to natural language, with the output being further processed into a CNN QSAR model to classify the sentences produced using natural language processing (NLP). Conversion of SMILES to graphical representation is known to be done to predict the relationships between a ligand and a protein [94]. This model uses protein sequences to construct a framework of interactions between the chemical and genomic space; hence a large amount of data is available for prediction. DeepACTION is a DTI prediction model that uses a novel technique called majority and minority instances balancing (MMIB) to balance a dataset between interacting and non-interacting pairs for an enhanced prediction [56]. QSAR modeling using SMILES has been used in other models as well [57]. Proteochemometrics (PCM) is an extension of QSAR models, and it uses both the ligand and the target descriptors to thoroughly map the compounds to its targets [78]. Unlike QSAR, PCM is multi-target processing and can pool related targets to increase the data available for learning. With a concept as such, it can provide information on the binding affinity measurements like inhibition constant (*K*_*i*_), dissociation constant (*K*_*d*_), or the half-maximal inhibitory concentration (IC_50_). DeepDTA is a PCM model developed with the help of only protein and SMILES strings data [58]. The CNN blocks performed better when the combination of sequences was fed. FRnet-DTI consists of two architectures, FRnet-Encode and FRnet-Predict. The former extracts 4096 features from gold standard datasets such as DrugBank, BRENDA, and KEGG; the latter classifies drug–protein interactions obtained from the features [59].

Assessing targets through compound sensitivity was attained using IC_50_ values present in the Genomics of Drug Sensitivity in Cancer (GDSC) database [60]. Using SMILES alongside this data, it is possible to predict IC_50_ values for any given compound. This model focuses on finding genes most relevant to the drug sensitivity prediction rather than the complete set of genes. DeepPurpose is another model that predicts IC_50_ values [61]. Another critical property considered in drug designing is the compound’s aqueous solubility. It can be predicted using SMILES codes that are analyzed on the CNN model ConvS2S [62]. A model DeepConv-DTI can predict drug–target interactions using only protein sequences, and it identifies local patterns important for target binding sites [63]. DTI-CNN, a network-based approach, constructs a heterogeneous network using data from different drug and protein-related sources to further identify DTIs, with a potential usage extending toward drug–drug and protein–protein interactions [64].

### Prediction of drug–drug interactions

In practical circumstances, more than one drug can be present in our body or consumed simultaneously to achieve an effect dissimilar to what is produced from individual drugs; effects that can be positive (synergistic) such as greater efficacy and reduced drug resistance or negative (antagonistic) such as increased toxicity, inhibitory, and other side effects. Drug–drug interactions (DDIs) elucidate such behaviors and are usually assessed during clinical trials to record such reactions. In a DDI task, both the subject to be studied and the object to be recognized is a drug, and machine learning-based methods observe this classification in two sections: is there an interaction? If yes, then the type of interaction.

DrugBank is a major database that includes DDI data [95]. MEDLINE is another essential database that consists of biomedical literature citing such interactions. A healthcare professional who wishes to identify an interaction between any two drug compounds must read through the entire literature before arriving at a conclusion. Another disadvantage of having such data is that this information cannot be used directly as an input to software as they are present as unstructured data in the literature. Extracting DDIs from such text manually is challenging as these databases are vast. NLP is a study that involves the use of artificial intelligence to extract meaningful information from human language and can be paired with traditional machine learning models [96], but these prove to be cumbersome as they require manual feature extraction [97]. Machine learning methods that use text learning detect the words around target drugs and evaluate tasks by identifying the exact words around drugs when required to predict unknown interactions. However, these models cannot recognize synonyms from the rest of the vocabulary without any external features and consider each word to have a unique definition [98]. Hence, there is a need to apply NLP with deep learning architectures that can detect essential features automatically.

Models with an NLP approach for DDI tasks must follow two steps: recognize the drug and relation extraction. One such method using NLP was given by Liu et al. that generated matrices of a concatenation of position and word embeddings of drugs of interest that appear in literature sentences [65]. CNNs are handy for designing solutions in this context as they can locate and track drugs’ positions in sentences. A dataset of DDIs could then be created using drug pairs identified in the same sentence, for example, ‘When drug1 is administered in combination with drug2’. The DDI corpus designed for the 2013 DDI Extraction challenge [99] is where several DDI models are trained and evaluated and consists of DDI pairs classified into five categories: mechanism (pharmacokinetic), advice (recommendation about DDI), effect (pharmacodynamic), interaction (Int), and false (no interaction). Int is a sentence containing a DDI pair and no other additional information, and false represents drug pairs that have no interaction between them. A multi-channel CNN was developed by Quan et al. that assigned different channels for different aspects of word embeddings [66].

Further down the timeline, CNN models recorded a similar accuracy without using any external features for classification, as demonstrated by Suárez-Paniagua et al. [67]. This was a significant step up, as deep learning could now be represented for what they are meant to be: a feature learning model. A two-stage learning process developed by the same group ran the eHealth-KD challenge dataset [100] and applied a bidirectional long short-term memory (Bi-LSTM) for drug recognition and a CNN for relation extraction [68]. A more recent architecture developed following NLP is a bidirectional gated recurrent unit—convolutional neural network (SGRU-CNN) hybrid model [69], while other hybrid models include attention-based graph convolutional network (AGCN) in 2020 by Park et al. [70] and recurrent hybrid convolutional neural network (RHCNN) in 2019 by Sun et al. [71]. Although CNNs and deep learning show immense promise, one of the limitations associated with deep learning models is that it follows a ‘black box’ approach [101], meaning it is hard to understand the mechanism from the results obtained.

## Conclusion and prospects

Various models in CNNs, as well as other deep learning architectures, have been developed to a large extent in the field of bioinformatics and have implications in pharmacogenomics. The massive parallelization is unmatched compared to sequence analysis using conventional experimentations; while the latter is accurate and reliable, the level of skill and human effort required to achieve such practices is limiting its pace. It is evident that statistical and machine learning models have brought about novel tools for analysis, and these technologies have significantly reduced the time and cost required. These models have now paved the way for newer deep learning models, like CNN, of higher complexity, assisted by increased data availability and computational power. With the increase in models’ complexities, however, more input features are required, as the model’s power relies on the amount of data. Inconsistencies in the data could also lead to failures in producing valid outputs. Altering the model architecture and fine-tuning parameters and hyperparameters to achieve maximum performance is still a challenge.

In this review, we have seen how the models presented worked accurately for a given dataset and solved the problem efficiently, but due to the heterogeneity of the data available today, achieving versatility of a model will always be a challenging task. It could be solved through improved algorithms for transfer learning, reducing the time it takes to build a model. As we head toward the future, we look at the simultaneous time-and-cost reduction in sequencing technologies and analysis tools. Deep learning models like CNNs can hold great potential in offering approaches other than the conventional statistical methods. The continuous increase in the complexity of models constructed and a greater amount of data availability would only encourage solving problems related to the genome mechanism. With a greater understanding of data, precise annotations can be assembled, crucial sites in our genome can be visualized better, and ultimately, drug compounds can be developed more precisely for a potent treatment. With the advent of open-source tools, the informatics community will lead the way for precision and personalized medicine accessible to everyone on this planet.
